# Supplementary material for: An assessment of prevalence of Type 1 CFI rare variants in European AMD, and why lack of broader genetic data hinders development of new treatments and healthcare access
Source: PLoS One. 2022 Sep 6;17(9):e0272260. doi: 10.1371/journal.pone.0272260 (PMC9447915; doi:10.1371/journal.pone.0272260)
Supplement: S1 Table — (DOCX) [file pone.0272260.s001.docx]

**S1 Table. Description of European AAMD and control datasets used to evaluate prevalence of Type 1 *CFI* rare variants.** AAMD; advanced age-related macular degeneration, FINBBB; Finnish Biobank Cooperative, IAMDGC; International AMD Genomics Consortium, PCA; principal component analysis, PheWAS; Phenome-wide association studies, NGS; next-generation sequencing, SNP; Single nucleotide polymorphism.

|  |  | **AAMD datasets** | | **Control datasets** | |
| --- | --- | --- | --- | --- | --- |
| **Study** | **Methods for variant identification** | **Method of disease ascertainment** | **Methods for determine ethnicity** | **Control source** | **Methods for determine ethnicity** |
| SCOPE | Targeted next-generation sequencing (NGS) | Retinal specialist confirmation of GA | Self-reported status | GnomAD non-Finnish European (v2.1.1) (38) | PCA analysis using genotype data |
| UK Biobank pheWAS portal (44) | Whole-exome sequencing | Self-reported disease status mapped to ICD10 code H35.3 | PCA analysis using genotype data | UK Biobank pheWAS portal (44) | PCA analysis using genotype data |
| IAMDGC (34) | Custom modified HumanCoreExome SNP array, with enriched protein-altering variants in AAMD risk genes identified in previous AAMD targeted NGS and whole genome sequencing studies (41,47) | Retinal specialist confirmation of any AAMD | PCA analysis using genotype data | IAMDGC (34) | PCA analysis using genotype data |
| FINBB | Targeted NGS | Health registry disease status mapped to ICD10 code H35.3 | None | GnomAD Finnish European (v2.1.1) (38) | PCA analysis using genotype data |
| Kavanagh et. al. (2015) (22) | Targeted NGS | Retinal specialist confirmation of any AAMD | Self-reported status | Kavanagh et. al. (2015) (22) | Self-reported status |
