# Supplementary material for: An assessment of prevalence of Type 1 CFI rare variants in European AMD, and why lack of broader genetic data hinders development of new treatments and healthcare access
Source: PLoS One. 2022 Sep 6;17(9):e0272260. doi: 10.1371/journal.pone.0272260 (PMC9447915; doi:10.1371/journal.pone.0272260)
Supplement: S2 Table — (DOCX) [file pone.0272260.s002.docx]

**S2 Table. Type 1 *CFI* rare variant frequencies in different European AAMD and control datasets.** ACMG; American College of Medical Genetics and Genomics, Chr; chromosome, FINBB; Finnish Biobank Cooperative, IAMDGC; International AMD Genomics Consortium, RAF; Rare allele frequency, UKB; UK Biobank, VUS; variant of uncertain significance.

| **Amino acid change** | **Functional Role** | **Chr** | **Position** | **Reference Allele** | **Rare Allele** | **ACMG classification** | **Kavanagh case RAF (n=2,266)** | **Kavanagh control RAF (n=1,400)** | **IAMDGC case RAF (n=16,144)** | **IAMDGC control RAF (n=17,832)** | **UKB case RAF (n=3,770)** | **UKB control RAF (n=234,829)** | **SCOPE case RAF (n=3,243)** | **FINBB case RAF (n=943)** |
| --- | --- | --- | --- | --- | --- | --- | --- | --- | --- | --- | --- | --- | --- | --- |
| p.Pro50Ala | missense | 4 | 110687890 | G | C | VUS | 0.088% | 0.000% | 0.000% | 0.000% | 0.000% | 0.000% | 0.185% | 0.000% |
| p.Pro64Leu | missense | 4 | 110687847 | G | A | VUS | 0.044% | 0.000% | 0.000% | 0.000% | 0.000% | 0.000% | 0.031% | 0.000% |
| p.Glu109Ala | missense | 4 | 110687712 | T | G | VUS | 0.044% | 0.000% | 0.000% | 0.000% | 0.000% | 0.000% | 0.000% | 0.000% |
| p.Gly119Arg | missense | 4 | 110685820 | C | T | Likely Pathogenic | 0.441% | 0.143% | 0.300% | 0.081% | 0.748% | 0.214% | 1.079% | 0.106% |
| p.Val152Met | missense | 4 | 110685721 | C | T | VUS | 0.088% | 0.000% | 0.053% | 0.006% | 0.027% | 0.026% | 0.247% | 0.000% |
| p.Gly162Asp | missense | 4 | 110682846 | C | T | VUS | 0.177% | 0.000% | 0.028% | 0.000% | 0.000% | 0.000% | 0.031% | 0.000% |
| p.Asn177Ile | missense | 4 | 110682801 | T | A | VUS | 0.044% | 0.000% | 0.000% | 0.000% | 0.000% | 0.000% | 0.031% | 0.000% |
| p.Val230Met | missense | 4 | 110681763 | G | A | VUS | 0.044% | 0.000% | 0.000% | 0.000% | 0.000% | 0.000% | 0.031% | 0.000% |
| p.Ala240Gly | missense | 4 | 110681732 | G | C | VUS | 0.530% | 0.071% | 0.000% | 0.000% | 0.000% | 0.000% | 0.524% | 0.000% |
| p.Ala258Thr | missense | 4 | 110681679 | C | T | VUS | 0.177% | 0.000% | 0.111% | 0.028% | 0.080% | 0.016% | 0.185% | 0.000% |
| p.Gly287Arg | missense | 4 | 110681450 | C | T | VUS | 0.132% | 0.000% | 0.111% | 0.028% | 0.159% | 0.066% | 0.093% | 0.000% |
| p.Asp310Glu | missense | 4 | 110673634 | C | A | VUS | 0.088% | 0.000% | 0.000% | 0.000% | 0.000% | 0.000% | 0.000% | 0.000% |
| p.His418Leu | missense | 4 | 110667554 | T | A | VUS | 0.088% | 0.000% | 0.000% | 0.000% | 0.000% | 0.000% | 0.031% | 0.000% |
| p.Cys467Arg | missense | 4 | 110667408 | A | G | VUS | 0.044% | 0.000% | 0.000% | 0.000% | 0.000% | 0.000% | 0.000% | 0.000% |
| p.Arg474Ter | stop-gained | 4 | 110667387 | G | A | Likely Pathogenic | 0.088% | 0.000% | 0.006% | 0.006% | 0.000% | 0.003% | 0.123% | 0.106% |
| p.Arg502Cys | missense | 4 | 110663677 | G | A | VUS | 0.044% | 0.000% | 0.000% | 0.000% | 0.000% | 0.000% | 0.000% | 0.000% |
| p.Val543Ala | missense | 4 | 110662173 | T | C | VUS | 0.044% | 0.000% | 0.000% | 0.000% | 0.000% | 0.000% | 0.000% | 0.000% |
| NA | splice donor | 4 | 110667377 | C | G | Pathogenic | 0.177% | 0.000% | 0.000% | 0.000% | 0.000% | 0.000% | 0.000% | 0.000% |
| **Sum=** |  |  |  |  |  |  | **2.383%** | **0.214%** | **0.610%** | **0.149%** | **1.014%** | **0.325%** | **2.590%** | **0.212%** |
