# Supplementary material for: An assessment of prevalence of Type 1 CFI rare variants in European AMD, and why lack of broader genetic data hinders development of new treatments and healthcare access
Source: PLoS One. 2022 Sep 6;17(9):e0272260. doi: 10.1371/journal.pone.0272260 (PMC9447915; doi:10.1371/journal.pone.0272260)
Supplement: S3 Table — (DOCX) [file pone.0272260.s003.docx]

**S3 Table. Type 1 *CFI* rare variant frequencies in different gnomAD populations.** Chr; chromosome, RAF; Rare allele frequency.

| **Amino acid change** | **Chr** | **Position** | **Reference Allele** | **Rare Allele** | **V2.1.1 European (Non-Finnish) RAF (n= 64,562)** | **V3.1.1 African/African-American RAF (n=20,744)** | **V2.1.1 Latino RAF (n=17,720)** | **V2.1.1 European (Finnish) RAF (n= 12,562)** | **V2.1.1 South Asian RAF (n=15,308)** | **V2.1.1 East Asian RAF (n=9,977)** | **V2.1.1 Ashkenazi Jewish RAF (n=5,185)** |
| --- | --- | --- | --- | --- | --- | --- | --- | --- | --- | --- | --- |
| p.Pro50Ala | 4 | 110687890 | G | C | 0.010% | 0.000% | 0.011% | 0.000% | 0.029% | 0.000% | 0.000% |
| p.Pro64Leu | 4 | 110687847 | G | A | 0.002% | 0.002% | 0.164% | 0.000% | 0.016% | 0.000% | 0.000% |
| p.Glu109Ala | 4 | 110687712 | T | G | 0.000% | 0.000% | 0.000% | 0.000% | 0.000% | 0.000% | 0.000% |
| p.Gly119Arg | 4 | 110685820 | C | T | 0.085% | 0.002% | 0.006% | 0.008% | 0.000% | 0.000% | 0.010% |
| p.Val152Met | 4 | 110685721 | C | T | 0.009% | 0.002% | 0.000% | 0.000% | 0.003% | 0.000% | 0.000% |
| p.Gly162Asp | 4 | 110682846 | C | T | 0.001% | 0.000% | 0.006% | 0.000% | 0.000% | 0.000% | 0.000% |
| p.Asn177Ile | 4 | 110682801 | T | A | 0.011% | 0.000% | 0.009% | 0.000% | 0.000% | 0.000% | 0.000% |
| p.Val230Met | 4 | 110681763 | G | A | 0.000% | 0.000% | 0.000% | 0.000% | 0.000% | 0.000% | 0.000% |
| p.Ala240Gly | 4 | 110681732 | G | C | 0.009% | 0.000% | 0.000% | 0.000% | 0.000% | 0.000% | 0.540% |
| p.Ala258Thr | 4 | 110681679 | C | T | 0.024% | 0.002% | 0.000% | 0.004% | 0.000% | 0.000% | 0.000% |
| p.Gly287Arg | 4 | 110681450 | C | T | 0.009% | 0.005% | 0.003% | 0.000% | 0.000% | 0.000% | 0.000% |
| p.Asp310Glu | 4 | 110673634 | C | A | 0.000% | 0.000% | 0.000% | 0.000% | 0.000% | 0.000% | 0.000% |
| p.His418Leu | 4 | 110667554 | T | A | 0.003% | 0.000% | 0.000% | 0.000% | 0.000% | 0.000% | 0.000% |
| p.Cys467Arg | 4 | 110667408 | A | G | 0.000% | 0.000% | 0.000% | 0.000% | 0.000% | 0.000% | 0.000% |
| p.Arg474Ter | 4 | 110667387 | G | A | 0.006% | 0.000% | 0.000% | 0.000% | 0.003% | 0.000% | 0.000% |
| p.Arg502Cys | 4 | 110663677 | G | A | 0.001% | 0.005% | 0.000% | 0.000% | 0.000% | 0.005% | 0.000% |
| p.Val543Ala | 4 | 110662173 | T | C | 0.000% | 0.000% | 0.000% | 0.000% | 0.000% | 0.000% | 0.000% |
| NA | 4 | 110667377 | C | G | 0.005% | 0.000% | 0.000% | 0.000% | 0.000% | 0.000% | 0.000% |
| **Sum=** |  |  |  |  | **0.174%** | **0.019%** | **0.198%** | **0.012%** | **0.052%** | **0.005%** | **0.550%** |
