# Supplementary material for: An assessment of prevalence of Type 1 CFI rare variants in European AMD, and why lack of broader genetic data hinders development of new treatments and healthcare access
Source: PLoS One. 2022 Sep 6;17(9):e0272260. doi: 10.1371/journal.pone.0272260 (PMC9447915; doi:10.1371/journal.pone.0272260)
Supplement: S4 Table — (DOCX) [file pone.0272260.s004.docx]

**S4 Table. Diversity of Type 1 *CFI* rare variant genotypes observed in background gnomAD populations.** RAF; Rare allele frequency.

| **GnomAD population** | **Number of different Type 1 *CFI* rare variant genotypes in background population (n, %)** |
| --- | --- |
| V2.1.1 European (Non-Finnish) RAF (n= 64,562) | 13 (72%) |
| V3.1.1 African/African-American RAF (n=20,744) | 6 (33%) |
| V2.1.1 Latino RAF (n=17,720) | 6 (33%) |
| V2.1.1 European (Finnish) RAF (n= 12,562) | 2 (11%) |
| V2.1.1 South Asian RAF (n=15,308) | 4 (22%) |
| V2.1.1 East Asian RAF (n=9,977) | 1 (6%) |
| V2.1.1 Ashkenazi Jewish RAF (n=5,185) | 2 (11%) |
